# 

**Published:** 1885-09

**Authors:** 


### BOOKS AND PAMPHLETS RECEIVED.

The Technology of Bacteria Investigation. By C. S. Dolley, M. D. Boston: S. E. Cassino & Co. 1885.

Von Ziemsein's Hand-Book of General Therapeutics. Volume II. New York: Wm. Wood & Co. 1885.

Laryngeal Hemorrhage. By J. W. Gleitsmann, M. D. New York: Reprint *American Journal of the Medical Sciences*, April, 1885.

Deviation of the Nasal Septum. By J. W. Gleitsmann, M. D. New York: Reprint *American Journal American Sciences*, July, 1885.

Some Interesting Reflex Neuroses. By J. J. Caldwell, M. D. Baltimore, Md.: Reprint *Virginia Medical Monthly*.

An Anomalous Human Lung, having four lobes on the right side. By W. A. Edwards, M. D. Reprint *American Journal Medical Sciences*, July, 1885.

A Memoir of Chas. Hilton Fagge, M. D. Printed for American distribution. By P. Blakiston, Son & Co. Philadelphia.

Second Report of the State Board of Health of the State of

Tennessee, October, 1880—December, 1884. J. Berrien Lindsey, M. D., Secretary. Nashville, Tenn.

COLLEGE ANNOUNCEMENTS:

Atlanta Medical College, 1885–86. Cincinnati College of Medicine and Surgery, 1885–86. Bellevue Hospital Medical College, 1885–86. Medical Department University of the city of New York, 1885–86. College Physicians and Surgeons, Baltimore, Md., 1885–86. Medical Department of the Tulane University of Louisiana, 1885–86. Medical Department University of Louisville, 1885–86. The New York Polyclinic, 1885. Baltimore Polyclinic and Post-Graduate Medical School, 1885. Miami Medical College, 1885–86. Medical College of Virginia, 1885–86. University of Maryland, 1885–86. Jefferson Medical College, 1885–86. The New York Post-Graduate Medical School and Hospital, 1885.

SHADOWS IN THE ETHICS OF THE INTERNATIONAL MEDICAL CONGRESS. By Levi Cooper Lane, M. D., M. R. C. S. San Francisco, California.

A CASE OF POISONING from Chloroform taken Internally—Recovery. By Llewellyn Eliot, M. D. Reprint from *Medical Record.*

VOICE OF SINGERS, read before the Ohio State Medical Society. By C. H. Von Klein, A. M., M. D. Dayton, Ohio.

POISONS, THEIR EFFECTS AND DETECTION. By Alex. Wynter Blyth, M. R. C. S. F. C. S., etc. Volume II. New York: Wm. Wood & Co. 1885.

ON RENAL AND URINARY AFFECTIONS. By W. Howship Dickinson, M. D. Cantab., F. R. C. P. New York: Wm. Wood & Co. 1885.

### OUR ADVERTISERS.

SEE advertisement of Medical College of Virginia, page 49, and write for catalogue.

THE special attention of physicians, and of all our readers, is invited to our advertising pages, where much will be found of interest to them. In writing to these advertisers, always mention THE ATLANTA MEDICAL AND SURGICAL JOURNAL.

MAGNUS & HIGHTOWER, ATLANTA.—It is a pleasure to commend to the profession this well-known drug house. We hazard nothing in saying that there is not a wholesale drug house in the South better prepared to supply the wants of physicians than this one. See their advertisement on page 6, and write them for prices.

NEW YORK POST GRADUATE SCHOOL.—We desire to call the attention of our readers to the advertisement of this well-known school for practitioners, to be found elsewhere. Write to Dr. C. L. Dana, 50 W. 46th street, for catalogue giving full particulars.

CINCINNATI COLLEGE OF MEDICINE AND SURGERY.—Attention is directed to the advertisement of this college, to be found at the top of page 29. Write to the dean at 164 George street, Cincinnati, for announcement, giving full particulars as to faculty, fees, etc.

HORSFORD'S ACID PHOSPHATE.—Of this well-known preparation, advertised elsewhere in THE JOURNAL, Dr. N. S. Read, Chicago, says: "I think it is a remedy of the highest value in many forms of mental and nervous exhaustion, attended by sick headache, dyspepsia and diminished vitality."

COLDEN'S LIQUID BEEF TONIC.—We take pleasure in calling the attention of our readers to the very attractive advertisement of this popular preparation, to be found on page 48. C. N. Crittenton, Sole Agent, 115 Fulton street, New York, will send samples to any regular physician writing for the same. See the advertisement, and if you are not prescribing it send for a sample bottle and give it a trial.

